# Z-Score Reference Values for Height in Turkish Children Aged 6 to 18 Years

**DOI:** 10.4274/Jcrpe.1260

**Published:** 2014-03-05

**Authors:** Hülya Günöz, Rüveyde Bundak, Andrzej Furman, Feyza Darendeliler, Nurçin Saka, Firdevs Baş, Olcay Neyzi

**Affiliations:** 1 İstanbul University Faculty of Medicine, Department of Pediatrics, İstanbul Turkey; 2 Boğaziçi University, Institute of Environmental Sciences, İstanbul, Turkey

**Keywords:** Z-score, children, Turkish

## Abstract

**Ob­jec­ti­ve**: Standard deviation score or Z-score reference charts are used in some countries in preference to percentile charts and are considered as better tools in assessing children with measurements outside the accepted limits of normality. Growth data for Istanbul children have previously been reported as percentiles; hence, the aim of this study is to present these data in Z-score reference tables. Data on secular trend in height in Turkish children will also be presented.

**Methods**: Height and weight data based on a total of 11 664 height and 11 655 weight measurements in 1100 boys and 1020 girls between 6 and 18 years of age obtained by biannual visits to schools were analyzed. All children came from well-to-do families and were all healthy. All measurements were made by two trained technicians. The LMS method was used in the analyses. The results were expressed as Z-score values for age.

**Results**: Heights of the boys and girls in all age groups were close to the updated USA growth references and showed an upward trend from previous data on Turkish children.

**Conclusions**: Height growth in Turkish school-age children of high socioeconomic level conforms to the updated growth data for USA children and also shows a secular trend. The data also point to the importance of updating local growth data periodically.

## INTRODUCTION

Percentiles and Z-score values for age are both useful indices in assessment of growth and either can be used as references. The important point is the availability of standard percentile or Z-score values to be used as references. Provided adequate statistical methods are applied to normalize the anthropometric growth data; Z-scores or standard deviation score (SDS) reference tables and charts are preferred by many researchers because these references, compared to percentiles, are more accurate in assessing children with measurements which lie outside the accepted limits of normality and they also facilitate further statistical analyses (1,2,3,4). Indeed, many countries use growth references constructed as Z-score charts or tables in preference to percentiles (5,6,7).

In recent years, a World Health Organization (WHO) study group has presented new growth charts for ages 0 to 60 months based on measurements of infants and children from eight different countries and has recommended the use of these charts as international references (8). For older age groups, WHO continues to recommend the use of growth charts developed by the National Center for Health Statistics (NCHS) for USA children in 1977 as international reference values (9,10). The WHO group emphasizes that data used for construction of reference growth standards should be based on measurements of children who not only are free of disease but are also reared in a healthy environment that minimizes constraints on growth such as poor diets and infection (8). This same principle was observed in the construction of growth charts for Turkish children prepared in 1978, which are in use in the country for many years (11,12,13,14,15). These charts represent weight and height values of a group of Istanbul children from the higher strata of the population, i. e. children who were healthy and relatively free of constraints faced by the lower socioeconomic groups in Turkey.

This present study was undertaken to update and improve the growth reference charts for Turkish children. The growth data, as before, were based on measurements of Istanbul children from well-to-do families. The percentile values obtained on this group were reported in previous publications (16,17). In this paper, Z-score values for height derived from the data on these same children will be presented.

## METHODS

The sample consisted of 1100 boys and 1020 girls between 6 and 18 years of age attending primary and secondary schools located in six different districts of Istanbul City. Students in these same schools had been assessed for growth in an earlier study (11,12,13). All six schools were located in relatively well-off districts. The data were collected between the years 1989 and 2002 by biannual visits to the schools by a team consisting of one pediatrician, two trained technicians and two physicians training in pediatrics. Using the school files, all children in one class at a time, whose birthdays were ±3 months from the prospective date of examination, were selected as subjects to be measured at the next visit. Information on the study and on the importance of height and weight measurements was given to the children in the groups. Written parental consent was obtained with the help of the school administration. Children who refused to cooperate were excluded. Children of younger ages (6-10 years) attending the first and second grades were included in the study and these same children were followed for 5 years until completion of the primary school (5 years at the time of the study). For the age groups 11-18 years, children in the first and second years of secondary schools were taken and to reach adequate numbers for each age group, inclusion of new subjects was continued for three consecutive years. Over time, measurements were repeated at six month intervals on these same children. Thus, our sample consists of a mixture of children followed longitudinally over different periods of time.

Chronological age was computed from the birth date reported by the child and verified by the school files. If these two sources disagreed, the child was not included in the study. Chronic or debilitating disease, assessed by history and a brief physical examination, was also a reason for exclusion.

Heights were measured in standing position with bare feet, using a portable measuring device (the Leicester height measure, Invicta Plastics, Ltd, UK). All measurements were performed by the same two trained technicians. Height measurements were repeated twice and the mean value was calculated. In case of a discrepancy exceeding 0.3 cm, a third measurement was done and the mean of the two closest values was used.

After all data were collected, the subjects were allocated to socioeconomic classes (SECs) by the same criteria used in our previous study (11,12,16,17). This arbitrary classification is based on the education level of both parents and the occupation of the fathers. The children had to meet all three criteria to be included in the higher levels of SECs; if not, assignment to a lower SEC resulted. In the occasional cases in which the qualifications did not agree with any of the groups described in the table, the student’s school file was reviewed and the child was included in the nearest suitable socioeconomic group. Since no significant differences were noted in height values between SECs 1 and 2 in the previous study or in this present study, children falling into both classes (SECs 1 and 2) were included in this present analysis. Dates of birth of the children ranged between 1974 and 1989.

The final data set used to derive the Z-score values is based on 6007 height measurements for boys and 5657 for girls. The mean number of measurements per child was 5.5±3.3. With the exception of age groups 6, 17.5 and 18 years, each half age group included measurements over 100 subjects.

Although the majority of the children were followed for different periods of time, longitudinal data covering ages 6 to 18 were not available for all children. The data were therefore analyzed cross-sectionally. The height data were smoothed as cubic splines by non-linear regression, using penalized likelihood with the program LMS (18,19). Since height-for-age values tend to be normally distributed, the SDS (Z-score) values were calculated using the equation (z=[measurement-mean]/SD).

## RESULTS

For each age and sex group, number of measurements, mean and SD values for height in both sexes and Z-score values computed from these values are given in Tables 1 and 2. In Table 3, present data are compared with mean, ±1 and ±2 values for boys and girls in the age groups 9 to 17 years born between the years 1950-1960 (11,12,13). The upward trend in height at almost all age groups can be noted.

## DISCUSSION

Assessment of growth is an essential part of pediatric health supervision at all ages and deviations from normal values usually implicate a pathologic condition. Height for age, expressed as centiles or SDS (Z-score) can be considered as the “golden standard” for the assessment of growth in children of school age and of pubertal years. An arrest in height growth can constitute the only sign of disease and the establishment of this deviation can be a valuable tool for early diagnosis. Particularly in communities where chronic malnutrition still prevails and diagnosis and accurate estimation of aberrations in the growth in individual children is of importance, Z-scores or SDS reference tables and charts provide a useful and practical index for growth assessment and follow-up of children, including those which show extreme deviations from the mean values.

This study presents height data obtained from a representative sample of Turkish children aged 6-18 years and adds Turkey to the list of countries which have updated the growth charts for their respective children. Taking into account the existing differences in height-for-age and weight-for-age values due to socioeconomic disparities, the growth charts of Turkish children presently in use in this country, as well as the updated percentile charts which were previously reported and the Z-score tables presented in this paper, were based on measurements of Istanbul children of well-to-do families (11,12,13,16,17). These references are therefore selective, but, in accordance with the recommendations of WHO, are representative of Turkish children who are born and reared in optimal or near optimal circumstances. It must be added that, as a result of a flow of emigration from all parts of the country during the past 60 years, 17% of the Turkish population now resides in Istanbul and we believe that the diverse composite population of Istanbul City is quite representative of the whole of Turkey (20).

The design of our study largely conforms to the criteria suggested by Waterlow and adopted by WHO, requiring that the reference population be well-nourished, the sampling procedure clearly defined and reproducible, the sample of adequate size, the measurements relevant and of good quality and the data adequately treated (8,9,10,21,22,23,24). Our sample consisted of measurements on a group of children who were followed over variable periods of time and therefore the numbers were not suitable for a longitudinal analysis. To be able to have adequate numbers in each age group, the data were treated cross-sectionally in the analysis. With the exception of age groups 6, 17.5 and 18 years, the number of measurements for each half age group appears to be adequate. We realize that the longitudinal element in our series constitutes a drawback. However, the 1977 NCHS charts which were accepted as the international standard by WHO were also derived from a population which contained a longitudinal element (25).

Our approach to construction of references is, similar to that of WHO, ‘prescriptive’, rather than ‘d escriptive’, since these references will serve as a tool for the diagnosis of inappropriate growth (1,22,23).

Mean values for height for age obtained in this study are compared with those of Turkish children born 30 years earlier and with CDC 2000 Growth Charts for white North American children (11,24,25). The present reference height values for Turkish boys and girls aged 6 to 18 years conformed quite well to the USA standards, the differences being around 0.5-1.0 cm at all ages and not exceeding 2 cm in any age group.

It is reported that stature is a genetically determined trait, with heritability estimated at approximately 80% (26). Several genome-wide association studies on stature have recently been published, mostly on Caucasian-based populations (27,28,29). Turkish children born in the years 1974-1989 were taller than children born in the years 1950-1960. While the effect of genetic make-up cannot be denied, this finding indicates that in Turkey, environmental factors are still at work continuing to affect growth and that even children of high socioeconomic groups, who are born to well-to-do and educated parents, continue to grow taller over time and final height is still a dynamic entity. Indeed, contrary to the generally accepted opinion that height of the population has stabilized in the industrial countries of Western Europe (30), recent publications from some of these countries show the continuation of a positive secular trend in height (5,6). Our data shows an increase in SD values with age. As expected, these values are highest at pubertal ages and more pronounced in the boys.

The growth reference values need to be re-examined every 5 to 10 years for populations with suspected large secular changes and every 15-20 years for those suspected of little change (7). The findings of our study indicate a continuing upward secular trend in height-for-age values in Turkish children and point, therefore, to a need to update the growth data every decade or so. Analysis of the data revealed that there was a general trend in height to increase with the year of birth. Children born in the later years tended to be a little taller than those born in the preceding years, even within this short range of years. Hence, it is warranted to refer to an upward trend, not just a simple shift between two points in time separated by 30 years.

In conclusion, we believe these updated data will, hopefully, meet the need expressed by many professionals working with Turkish children in and outside Turkey (31,32) and will be of help in their assessment of the growth of individual children and in the diagnosis of growth problems.

## Figures and Tables

**Table 1:**
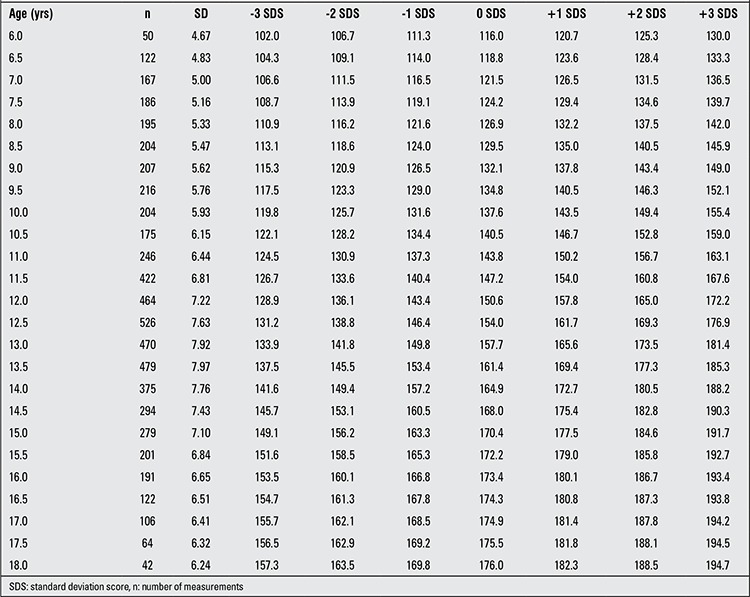
SD values, mean values (0 SDS) and values corresponding to Z-scores (±1, ±2, ±3 SDS) for height in Turkish boys aged 6 to 18 years

**Table 2:**
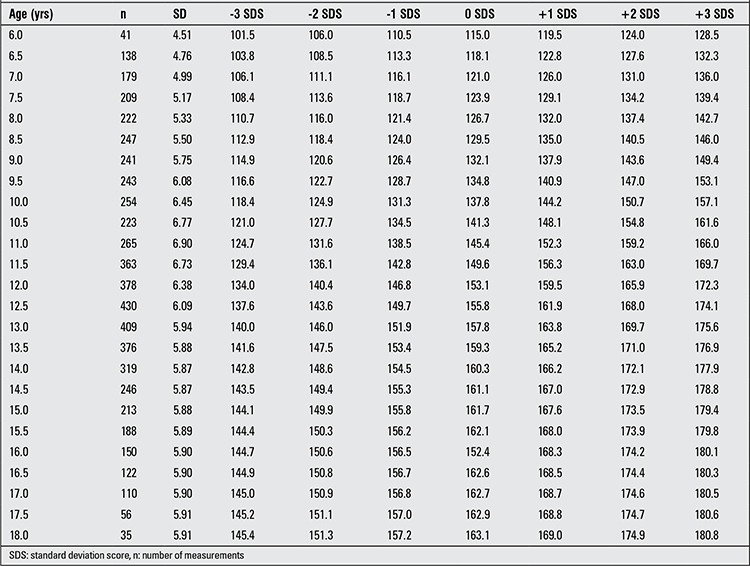
SD values, mean values (0 SDS) and values corresponding to Z-scores (±1, ±2, ±3 SDS) for height in Turkish girls aged 6 to 18 years

**Table 3:**
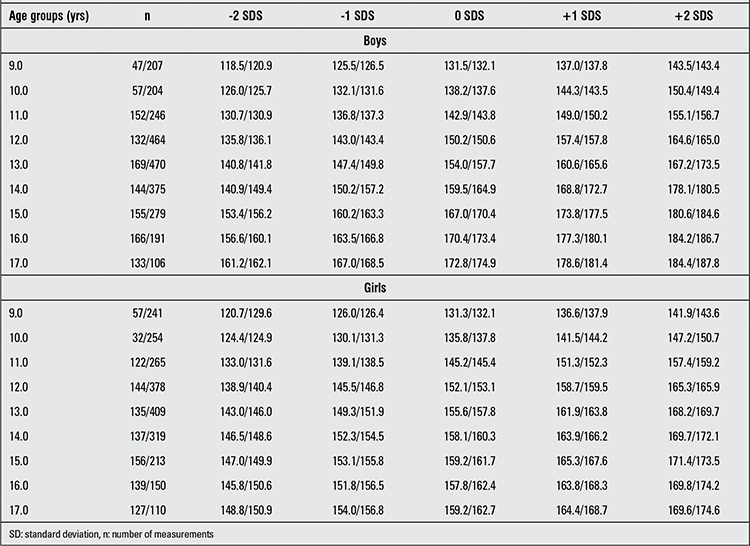
Heights (mean values and those corresponding to ±1 SD and ±2 SD values, in centimeters) in İstanbul children born in the years 1950-1960 and 1974-1989 [ages 9 to 17, group 1 (1950-1960 born)/group 2 (1974-89 born)]
